# Multifaceted Pollutant Removal by *Salicornia brachiata*: A Phytoremediation Approach

**DOI:** 10.3390/plants14131963

**Published:** 2025-06-26

**Authors:** Piyoni Ruwanpathirana, Imalshi Gunawardana, Hasini Navodya, Ajith C. Herath, Dinum Perera, Manavi S. Ekanayake

**Affiliations:** 1 Department of Bioprocess Technology, Faculty of Technology, Rajarata University of Sri Lanka, Mihintale 50300, Sri Lanka; sandunika042@gmail.com (P.R.); bst1819030@tec.rjt.ac.lk (I.G.); h.navodya.m@gmail.com (H.N.); 2 Department of Chemical Sciences, Faculty of Applied Sciences, Rajarata University of Sri Lanka, Mihintale 50300, Sri Lanka; 3 Department of Aquaculture and Fisheries, Faculty of Livestock, Fisheries and Nutrition, Wayamba University of Sri Lanka, Makandura 60170, Sri Lanka

**Keywords:** phytoextraction, halophyte, metals of toxicological concern, antioxidant enzymes, abiotic stress

## Abstract

The increasing discharge of nutrient and metal-laden effluents into saline environments demands sustainable remediation strategies. This study evaluated the phytoremediation potential of *Salicornia brachiata*, a halophytic plant, under hydroponic conditions using varying concentrations of three macronutrients—nitrate (NO_3_^−^), phosphate (PO_4_^3−^), and calcium (Ca^2+^)—and three heavy metals—lead (Pb^2+^), chromium (Cr^6+^), and copper (Cu^2+^). The plant exhibited high removal efficiencies across all treatments, with Pb^2+^ and Cr^6+^ reaching nearly 99% removal within two days, while macronutrient removal showed a steady, time-dependent increase over the 14-day period. Several biochemical parameters, including proline content and antioxidant enzyme activities (catalase, superoxide dismutase, peroxidase, polyphenol oxidase), were significantly affected by treatments, with most showing dose-dependent responses to heavy metal exposure, indicating strong biochemical resilience. Fourier transform infrared spectroscopy revealed pollutant-specific structural shifts and identified –OH, –NH, and –COO^−^ groups as key binding sites. The study quantifies the removal efficiency of *S. brachiata* for both nutrients and metals and provides mechanistic insight into its ionic stress response and binding pathways. These findings establish *S. brachiata* as a viable candidate for integrated phytoremediation in saline, contaminated water systems.

## 1. Introduction

Coastal ecosystems—including salt marshes, estuaries, seagrass beds, and mangroves—are ecologically vital transitional zones between land and sea. They provide essential services such as shoreline stabilization, carbon sequestration, nutrient cycling, and habitats for a diverse range of flora and fauna [1]. The global escalation of industrial activities, urbanization, and agricultural intensification has led to the discharge of various heavy metals, organic compounds, and excess nutrients into the environment, resulting in the accumulation of these pollutants in the water and sediments [2,3]. The complex interactions among tidal cycles, sediment dynamics, and vegetation make coastal ecosystems highly susceptible to pollutant accumulation, disrupting the delicate balance of aquatic life and posing significant risks to human health through the food chain [4,5,6].

Nitrates (NO_3_^−^) and phosphates (PO_4_^3−^), the essential nutrients for the growth of plants and algae, can act as pollutants when present in excessive amounts [3,7]. These excess nutrients, primarily originating from anthropogenic activities such as agricultural runoff and wastewater dischargers by municipal, industrial, or aquaculture practices, contribute to eutrophication that results in algal blooms, hypoxic dead zones, and overall degradation of ecosystem health [3,8]. Elevated calcium concentrations in aquatic environments can arise from anthropogenic sources such as liming and mineral supplementation in aquaculture and agricultural practices or through natural processes such as geological weathering and saline intrusion [9,10]. These changes can potentially alter ionic balance, water hardness, and nutrient dynamics in sensitive ecosystems [11,12]. Furthermore, anthropogenic activities release various heavy metals such as lead (Pb^2+^), chromium (Cr^6+^), and copper (Cu^2+^) into the environment, posing significant ecological risks due to their toxicity, persistence, and biomagnification potential [13]. These metals can disrupt physiological processes, impair growth and reproduction, and even cause mortality in aquatic species [13,14].

Managing pollutants at their source presents numerous challenges due to the dispersed nature of many pollutants, the high costs associated with advanced treatment technologies, and the lack of comprehensive regulatory frameworks in many regions [3]. Consequently, there is a growing need for innovative, cost-effective, and environmentally friendly approaches to mitigate pollutant impacts in coastal ecosystems. Phytoremediation, the use of plants to remove, degrade, or stabilize environmental contaminants, has emerged as a promising approach for addressing pollution in contaminated sites by harnessing plants’ natural ability to extract, sequester, or detoxify pollutants [15,16,17].

Halophytes, plants naturally adapted to saline environments, have significant potential for phytoremediation in coastal areas due to their remarkable tolerance to high salt concentrations and various environmental stressors. These plants employ diverse physiological and biochemical strategies such as ion compartmentalization, osmotic adjustment, succulence, selective ion transport, antioxidant activities, redox and energy balance maintenance, salt inclusion or excretion, and genetic regulation to survive under extreme conditions [18,19]. Compared to salt-sensitive plant species used in phytoremediation, halophytes are inherently more resilient to environmental stressors, including heavy metals [20]. Among them, *Salicornia* species are particularly notable for their ability to tolerate and accumulate elevated levels of pollutants, making them promising candidates for use in environmental remediation [21,22].

Despite previous phytoremediation studies on various *Salicornia* species, the potential of *Salicornia brachiata* to remediate excess nutrients—nitrate (NO_3_^−^), phosphate (PO_4_^3−^), calcium (Ca^2+^)—and metals of toxicological concern such as lead (Pb^2+^), chromium (Cr^6+^), and copper (Cu^2+^) under controlled hydroponic conditions remains underexplored. Existing research has primarily focused on bioaccumulation trends, with limited emphasis on the temporal dynamics of pollutant removal, an essential parameter in assessing the plant’s applicability in real-world wastewater treatment systems and for optimizing phytoremediation strategies [22,23]. Furthermore, pollutant-specific interactions with plant biochemical functional groups such as hydroxyl, carboxyl, and amine moieties have not been adequately studied, limiting the understanding of contaminant binding and stress response mechanisms.

This study addresses these knowledge gaps by quantifying time-resolved removal efficiencies, assessing elemental accumulation, and characterizing plant responses through growth metrics, proline content, and antioxidant enzyme activity (catalase, peroxidase, superoxide dismutase, polyphenol oxidase). Additionally, structural alterations induced by specific pollutants are investigated to gain molecular-level insights into the interactions between contaminants and plant functional groups, thereby elucidating pathways underlying the *S. brachiata*’s pollutants detoxification mechanisms.

By extending the approach to aquaponic-compatible systems, the findings offer a low-cost, integrated phytoremediation strategy that promotes circular resource use and supports key United Nations Sustainable Development Goals (SDGs)—namely SDG 12 (Responsible Consumption and Production), SDG 6 (Clean Water and Sanitation), and SDG 14 (Life Below Water)—through sustainable water reuse and aquatic pollution mitigation.

## 2. Results and Discussion

### 2.1. Removal Efficiency of Nutrients and Heavy Metals by S. brachiata

**Figure 1:**
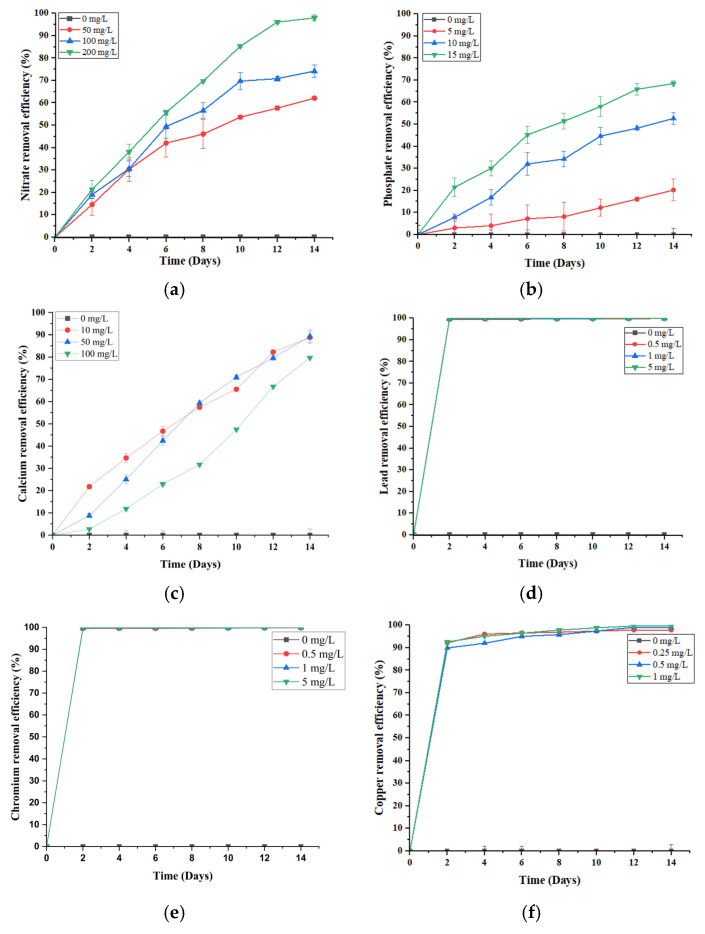
Removal efficiencies of (**a**) 0, 50, 100, 200 mg/L initial NO_3_^−^, (**b**) 0, 5, 10, 15 mg/L initial PO_4_^3−^, (**c**) 0, 10, 50, 100 mg/L initial Ca^2+^, (**d**) 0, 0.5, 1, 5 mg/L initial Pb^2+^, (**e**) 0, 0.5, 1, 5 mg/L initial Cr^6+^, and (**f**) 0, 0.25, 0.5, 1 mg/L initial Cu^2+^ by *S. brachiata* grown in aqueous solutions for 14 days. Error bars depict the standard errors of the means (n = 3); when error bars are not shown, the standard deviation was less than the width of the symbol.

Pollutant removal capacity remains a primary benchmark for assessing the effectiveness of a phytoremediation system. In this study, *S. brachiata* consistently demonstrated high removal efficiencies for nitrate (NO_3_^−^), phosphate (PO_4_^3−^), calcium (Ca^2+^), lead (Pb^2+^), chromium (Cr^6+^), and copper (Cu^2+^) over a 14-day hydroponic exposure, as illustrated in Figure 1.

The NO_3_^−^ removal efficiency of *S. brachiata* increased progressively over time across all concentrations (50, 100, and 200 mg/L), peaking at 96.06 ± 0.79% in the 200 mg/L treatment group by day 12 (Figure 1a). With a half-life reached by day 6 (55.76% removal from 200 mg/L of initial NO_3_^−^ concentration), this rapid uptake suggests the involvement of high-affinity NO_3_^−^ transporters operating effectively under moderate to high NO_3_^−^ availability. The sustained removal further indicates internal assimilation or compartmentalization into nitrogenous compounds, such as amino acids or osmolytes, contributing to the plant’s salinity tolerance.

Comparable NO_3_^−^ uptake efficiency has been well-documented in other *Salicornia* species under saline and nutrient-rich conditions. For instance, *Salicornia europaea* demonstrated enhanced NO_3_^−^ assimilation and biomass accumulation under combined NO_3_^−^ and NaCl treatments [24]. Similarly, *Salicornia persica*, used as a biofilter in constructed wetlands treating aquaculture effluents, achieved total nitrogen removal efficiencies of 100% and 81% under surface and subsurface flow systems, respectively, across both high (3.3) and low (0.13) nutrient loading rates (g N/m^2^/d) over a six-month period [25].

The robust NO_3_^−^ removal capacity demonstrated by *S. brachiata* in the current study, combined with evidence from related species, highlights the genus *Salicornia* as a promising candidate for the treatment of nitrogen-enriched saline effluents. Its rapid uptake kinetics, tolerance to salinity and high nutrient loads, and sustained physiological health position it to be well suited for use in phytoremediation strategies where NO_3_^−^ pollution is a major concern.

PO_4_^3−^ removal followed a similar concentration-dependent pattern, with efficiency increasing from 20.21% (5 mg/L) to 68.46% (15 mg/L) (Figure 1b). This enhanced uptake may be driven by stronger concentration gradients, facilitating both passive diffusion and active transport across root membranes, alongside the upregulation of high-affinity PO_4_^3−^ transporters [26,27]. As phosphorus is crucial for ATP production and cellular biosynthesis, moderate enrichment likely stimulates metabolic activity and incorporation into biomass [27,28].

Comparable responses have been reported in *Salicornia* spp., where PO_4_^3−^ uptake efficiency increased under moderate to high nutrient loads, particularly in saline conditions [29]. For instance, *S. persica* exhibited significantly higher PO_4_^3−^ uptake at 1.0 and 1.5 mM compared to 0.1 mM, especially under 0–200 mM NaCl salinity, while uptake declined beyond this threshold, indicating that extreme salinity can hinder membrane transport processes [30].

Beyond controlled greenhouse experiments, *Salicornia* species have also proven to be effective in pilot-scale recirculating aquaculture systems (RAS) [25]. For example, constructed wetlands planted with *S. europaea* achieved nitrogen removal efficiencies up to 98.2 ± 2.2% under ambient dissolved inorganic nitrogen loads (109–383 μmol/L), primarily in the form of ammonium and nitrate [31]. During routine operations, the same species also demonstrated effective removal of PO_4_^3−^, ranging from 36% to 89% [31]. Although phosphorus uptake is more variable due to its low mobility in saline media, these findings highlight *Salicornia*’s dual capacity to manage nitrogen and phosphorus pollution under real-world conditions [32].

The expansion of aquaculture and agriculture in coastal areas has contributed to elevated nutrient loads—particularly NO_3_^−^ and PO_4_^3−^—in adjacent water bodies, often leading to eutrophication, algal blooms, hypoxia, and biodiversity loss [3]. Halophytes such as *Salicornia*, with their high salt tolerance, fast growth, and nutrient bioaccumulation capacity, are ideal for use in constructed wetlands and integrated aquaculture systems [33]. Their dual function as biofilters and biomass crops offers a sustainable approach to mitigating nutrient pollution in saline and brackish environments [31,34].

In contrast to the concentration-dependent patterns observed for NO_3_^−^ and PO_4_^3−^, Ca^2+^ removal followed an inverse trend. The highest efficiency (89.3%) was recorded at the lowest initial concentration (50 mg/L), with a decline at 100 mg/L (79.67%) (Figure 1c). This suggests possible transporter saturation or physiological regulation of Ca^2+^ uptake under higher concentrations. Unlike NO_3_^−^ and PO_4_^3−^, which are actively absorbed and assimilated into key metabolic pathways, Ca^2+^ is primarily taken up passively via apoplastic flow and regulated based on cellular demand [35]. At lower external levels, uptake may be more efficient, supporting structural functions such as cell wall stabilization and ion signaling [35,36]. However, excess Ca^2+^—especially under saline conditions where Na^+^ competes for transport sites—can reduce uptake efficiency by altering membrane integrity or transporter activity [30,37].

Given that Ca^2+^ overaccumulation—resulting from liming, mineral leaching, and feed inputs—is a recognized concern in coastal aquaculture systems [11], the ability of *S. brachiata* to mitigate excess Ca^2+^ highlights its practical phytoremediation potential. Beyond water purification, its biomass offers added value as a gourmet food, forage, or bioenergy source, supporting integrated sustainability in coastal resource management [38].

In this study, macronutrients—nitrate (NO_3_^−^), phosphate (PO_4_^3−^), and calcium (Ca^2+^)—displayed a steady, time-dependent increase in removal efficiency over the 14-day period, while the heavy metals—lead (Pb^2+^), chromium (Cr^6+^), and copper (Cu^2+^)—exhibited a distinctly different pattern (Figure 1d–f). All heavy metals showed a rapid rise in removal efficiency within the first two days, irrespective of the initial concentration. Notably, Pb^2+^ (Figure 1d) and Cr^6+^ (Figure 1e) reached near-complete removal (~99%) by day two, followed by a plateau. Cu^2+^ removal (Figure 1f) was slightly slower, attaining ~90% by day 2 and gradually rising to ~99% by day 12.

This contrast in removal efficiencies over time highlights the divergent uptake mechanisms between macronutrients removal, i.e., nitrate (NO_3_^−^) and phosphate (PO_4_^3−^), which is largely driven by active transport and metabolic assimilation into compounds like amino acids and nucleotides [39], while Ca^2+^ a macronutrient is also primarily absorbed through passive apoplastic flow and cation exchange processes; however, its uptake can exhibit a similarly time-dependent trend due to sustained transpiration-driven flow and progressive binding within root tissues [35]. In contrast, metal removal appears dominated by passive biosorption and surface adsorption. *Salicornia* species possess mucilage layers and negatively charged cell walls that facilitate metal ion binding through ion exchange, complexation, and precipitation [20]. The rapid initial removal of Pb^2+^ and Cr^6+^ suggests surface-level immobilization without significant internal transport, as reported in other halophytes [20,40,41].

Cu^2+^, although initially adsorbed, likely undergoes additional internalization due to its essential role as a micronutrient. It can be taken up via COPT transporters, translocated, and incorporated into metalloproteins or chelated by phytochelatins [42], suggesting a dual mechanism of uptake and detoxification.

Overall, these findings indicate that *S. brachiata* employs two complementary strategies: rapid, surface-level exclusion of toxic metals and slower, metabolic assimilation of nutrients. This functional differentiation underscores its suitability for treating saline wastewater contaminated with both nutrient and heavy metal pollutants.

Although previous studies have demonstrated the capacity of the *Salicornia* species to accumulate metals such as Zn, Pb, Ni, Cd, and Cu under soil-based conditions [21,23,43,44], they have largely focused on tissue concentrations rather than removal efficiency from the medium. While *S. brachiata* has been reported to absorb Cd^2+^, Ni^2+^, and As^3+^ in hydroponics [22], few studies have quantified its removal kinetics. This study bridges that gap by providing one of the first detailed evaluations of heavy metal removal efficiency by *S. brachiata* under controlled hydroponic conditions.

### 2.2. Plant Growth Parameters and Biochemical Responses

The growth parameters (fresh weight, shoot length, number of lateral branches, and root length) and biochemical markers (chlorophyll, proline, catalase, superoxide dismutase [SOD], peroxidase [POD], and polyphenol oxidase [PPO]) of *S. brachiata* seedlings following 14 days of exposure to the selected excess nutrients and metals are summarized in Table 1, Table 2, Table 3, Table 4, Table 5 and Table 6. Overall, most growth parameters—namely fresh weight, shoot length, number of lateral branches, and root length—did not show consistent positive or negative trends across the full concentration ranges of nutrients and heavy metals. This variability likely reflects the halophytic nature of *S. brachiata*, which is known for its ability to buffer short-term physiological stress. However, specific parameters did exhibit significant trends in response to certain treatments.

For instance, fresh weight increased significantly with rising nitrate (NO_3_^−^) concentrations (0, 50, 100, and 200 mg/L; Table 1), highlighting NO_3_^−^’s central role as a macronutrient that enhances biomass accumulation. In salt-tolerant species such as *Salicornia*, moderate NO_3_^−^ enrichment can promote photosynthetic activity, osmolyte production, and nitrogen assimilation [45,46,47].

In contrast, shoot length decreased significantly with increasing copper (Cu^2+^) concentrations (0, 0.25, 0.5, and 1 mg/L; Table 6), consistent with Cu-induced toxicity. Excess Cu is known to cause oxidative stress, impair cell elongation, and disrupt hormonal regulation of shoot growth [42]. In species such as *S. europaea* and *S. brachiata*, Cu exposure has been associated with reduced apical growth, likely due to interference with auxin transport and suppression of meristematic activity [48,49].

In contrast to the variable growth trends, many biochemical parameters—especially antioxidant enzyme activities—showed clearer, dose-dependent responses to pollutant exposure. While chlorophyll content did not consistently follow a single trend, it was generally higher in control treatments across most stress conditions, except under phosphate (PO_4_^3−^) exposure. The observed chlorophyll reduction may stem from disrupted uptake of essential cofactors for chlorophyll synthesis, such as Mg^2+^, Fe^2+^, and Zn^2+^—a pattern consistent with findings in halophytes such as *S. europaea*, where metal stress led to oxidative damage and impaired nutrient assimilation [50,51,52].

Furthermore, heavy metals can impair chlorophyll synthesis by inhibiting key enzymes such as δ-aminolevulinic acid dehydratase (ALAD), which is essential in the biosynthetic pathway [53]. Previous studies on *Atriplex halimus* and *Suaeda maritima* have reported ALAD inhibition and chlorophyll decline under Pb^2+^, Zn^2+^, and Cd^2+^ exposure [54,55]. Similar findings in *S. brachiata*, and *S. europaea* show that metal-induced oxidative stress, ionic toxicity, and ROS-mediated lipid peroxidation disrupt chlorophyll metabolism and reduce pigment levels [22,50].

Despite the observed reduction in chlorophyll content, *S. brachiata* seedlings remained visibly green throughout the experimental period, with no signs of chlorosis or necrosis. This observation aligns with findings in *Salicornia iranica*, where chlorophyll declined under Pb^2+^ exposure without visible chlorosis, and in *S. europaea* and *A. halimus*, which retained green pigmentation under salt and metal stress due to sustained carotenoids and elevated peroxidase activity protecting chloroplast membranes [54,56]. Moreover, halophytes often engage in ion compartmentalization—sequestering toxic ions such as Pb^2+^, Cu^2+^, and Cr^6+^ into vacuoles or older tissues—which minimizes their interference with chlorophyll biosynthesis and function in actively photosynthesizing cells [57]. Such tolerance suggests that *Salicornia* species possess robust physiological mechanisms that mitigate the visual and functional symptoms of chlorophyll degradation under heavy metal stress.

Exposure to all tested pollutants—nitrate (NO_3_^−^), phosphate (PO_4_^3−^), calcium (Ca^2+^), lead (Pb^2+^), chromium (Cr^6+^), and copper (Cu^2+^)—resulted in elevated proline content in *S. brachiata*, underscoring the plant’s robust osmotic adjustment capacity under stress. In several treatments, proline levels increased progressively with rising pollutant concentrations, while in others, values remained significantly above the control, regardless of the dose. This response is consistent with findings in other *Salicornia* species. For example, *S. persica* and *S. brachiata* have both shown increased proline levels under salt and heavy metal stress, functioning to stabilize proteins, maintain membrane integrity, and scavenge reactive oxygen species (ROS) [22,51]. These results confirm that proline acts as a key biochemical marker of stress resilience in halophytes [51].

Similarly, the activities of antioxidant enzymes—catalase (CAT), superoxide dismutase (SOD), peroxidase (POD), and polyphenol oxidase (PPO)—were upregulated across most pollutant treatments in the present study. These increases were observed either as consistent positive trends across all tested concentrations or as marked enhancements compared to the control. This enzymatic activation is indicative of an oxidative stress response and mirrors earlier reports in *Salicornia* species. For instance, *Salicornia fruticosa* exposed to Cd^2+^ under saline hydroponic conditions exhibited elevated SOD, CAT, and ascorbate peroxidase activities, reinforcing the role of these enzymes in detoxifying ROS generated by metal stress [44]. Similarly, *S. brachiata* demonstrated increased CAT and POD activity under Ni^2+^, Cd^2+^, and As^3+^ exposure, suggesting that antioxidant regulation is a conserved stress-adaptive strategy in this genus [22].

Interestingly, phosphate (PO_4_^3−^) treatment in this study did not induce changes in antioxidant enzyme activity compared to the control (Table 1). This could be attributed to the essential role of phosphorus as a macronutrient, which is unlikely to provoke oxidative stress at the concentrations tested. Comparable trends have been observed in *S. europaea* grown in eutrophic waters, where phosphorus supplementation did not elicit a strong antioxidant response [31].

Notably, a deviation from the general antioxidant trend was observed in Pb^2+^ treatments, where SOD activity was undetectable at higher Pb^2+^ concentrations (Table 4). This suggests that Pb^2+^ may directly inhibit SOD synthesis or activity, likely due to its strong affinity for binding enzyme cofactors such as Zn^2+^ and Cu^2+^, which are essential for SOD function. Similar findings were reported in several non-halophytic plant species exposed to Pb^2+^, where higher Pb accumulation was associated with reduced SOD activity and increased lipid peroxidation, highlighting Pb’s disruptive impact on enzymatic antioxidant defenses [58,59].

Collectively, these findings demonstrate that *S. brachiata* responds to a range of pollutant-induced stresses with both osmoprotective (proline) and antioxidative biochemical strategies, consistent with patterns reported in other *Salicornia* species. However, the magnitude and specificity of responses appear to be pollutant and dose-dependent, revealing the nuanced physiological plasticity of halophytes under complex environmental stress conditions. This integrative response may contribute to the resilience and utility of *Salicornia* in phytoremediation applications, particularly in saline and pollutant-laden environments.

### 2.3. FTIR Analysis of S. brachiata Plants

**Figure 2:**
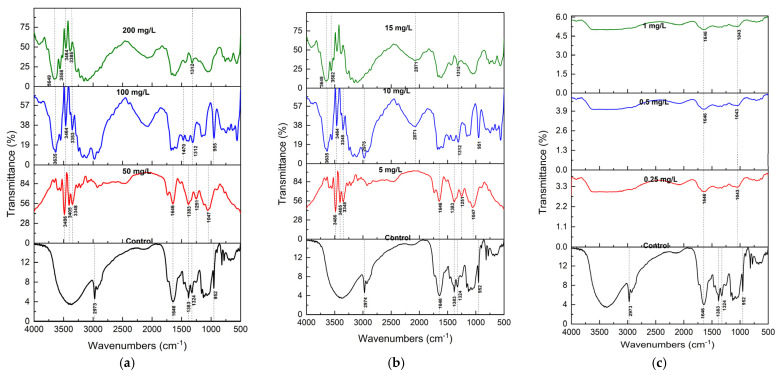
FTIR spectra (mean of n = 3) of *S. brachiata* plant samples exposed to (**a**) 0, 50 mg/L, 100 mg/L, 200 mg/L initial NO_3_^−^, (**b**) 0, 5 mg/L, 10 mg/L, 15 mg/L of initial PO_4_^3−^, and (**c**) 0, 0.5 mg/L, 1 mg/L, 5 mg/L of initial Cr^6+^ concentrations.

**Figure 3:**
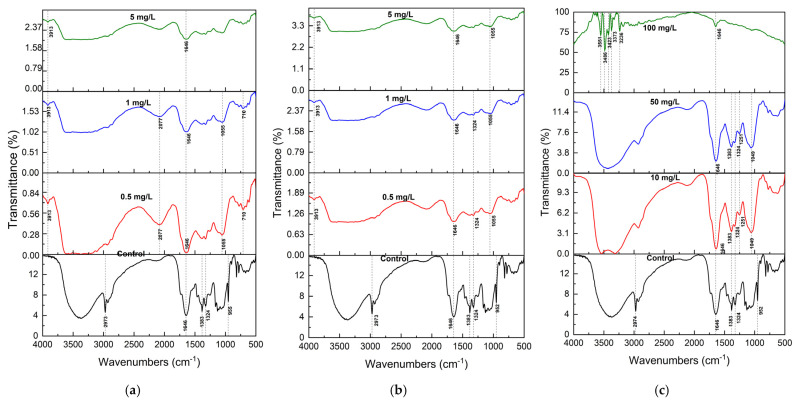
FTIR spectra (mean of n = 3) of *S. brachiata* plant samples exposed to (**a**) 0, 0.5 mg/L, 1 mg/L, 5 mg/L initial Pb^2+^, (**b**) 0, 0.25 mg/L, 0.5 mg/L, 1 mg/L of initial Cu^2+^, and (**c**) 0, 10 mg/L, 50 mg/L, 100 mg/L of initial Ca^2+^ concentrations.

Fourier Transform Infrared (FTIR) spectroscopy was employed to analyze the functional group alterations in *S. brachiata* tissues before and after 14-day exposure to selected pollutants, such as phosphate (NO_3_^−^), phosphate (PO_4_^3−^), chromium (Cr^6+^), lead (Pb^2+^), copper (Cu^2+^), and calcium (Ca^2+^) (Figure 2a and Figure 3c). The spectral changes provided insights into the molecular interactions underpinning pollutant adsorption and revealed pollutant-specific binding mechanisms.

In control samples, the FTIR spectrum displayed characteristic features of plant biochemical constituents. A broad band centered at 3350 cm^−1^ was attributed to O–H and N–H stretching vibrations, indicative of hydroxyl and amine groups present in carbohydrates, proteins, and phenolic compounds [60]. The peak at 2973 cm^−1^ corresponded to asymmetric C–H stretching in lipids [61], while a prominent band at 1646 cm^−1^ represented C=O stretching in the amide I bonds of proteins [62]. Additional bands at 1400 cm^−1^ and 1055 cm^−1^ were assigned to symmetric COO^−^ stretching in carboxylic acids and C–O stretching in alcohols and polysaccharides, respectively [63]. Bands at 1383, 1324, and 952 cm^−1^ were attributed to C=C, C–H, and C–O–C vibrations in polysaccharide structures [64,65].

Following exposure to NO_3_^−^ and PO_4_^3−^, the spectra showed significant broadening and the appearance of new peaks in the 3350 cm^−1^ region (Figure 2a,b). These alterations suggest strong hydrogen bonding and electrostatic interactions between anionic pollutants and protonated amino groups or hydroxyl moieties on the biomass surface. Such binding likely occurs via ion exchange and H-bonding to functional groups such as –NH_3_^+^ and –OH [66].

Cr^6+^ exposure resulted in a more ill-defined FTIR spectrum, with the disappearance of characteristic peaks in the 1300–500 cm^−1^ region. These bands are typically associated with alcohols and carboxylic acid groups. At pH ~7.0, Cr^6+^ exists primarily as HCrO_4_^−^ and CrO_4_^2−^ [67], and its uptake may involve not only adsorption but also redox transformations. The loss of O–H-related bands suggests that Cr^6+^ may be reduced to Cr^3+^ via oxidation of hydroxyl groups, consistent with previous findings in Cr^6+^ biosorption systems [68].

For cationic pollutants, including Pb^2+^, Cu^2+^, and Ca^2+^, FTIR spectra confirmed the involvement of hydroxyl and carboxylate functional groups in metal binding (Figure 3a–c). These groups are known to form chelates and dicarboxylate complexes with metal ions. The disappearance of the 955 cm^−1^ peak across all metal treatments indicates significant perturbation of polysaccharide or alcohol-linked structures. Notably, a new peak at 3913 cm^−1^ was observed in Pb^2+^- and Cu^2+^-treated samples but was absent in the Ca^2+^ spectrum, indicating ion-specific interactions, possibly due to differences in hydration behavior and complexation tendency.

Although these metal ions have similar ionic radii (Pb^2+^: 0.119 nm; Cu^2+^: 0.073 nm; Ca^2+^: 0.112 nm), their hydration free energies differ significantly. Pb^2+^, with the lowest hydration energy (−1492 kJ/mol), is more readily dehydrated and thus more likely to bind to biomass surfaces than Cu^2+^ (−2076 kJ/mol) or Ca^2+^ (−1588 kJ/mol) [69,70]. This explains the observed thermodynamic preference for Pb^2+^ adsorption in *S. brachiata.*

Overall, FTIR analysis demonstrated pollutant-specific spectral modifications and confirmed that *S. brachiata* biomass contains a wide array of active functional groups—including hydroxyl, carboxylate, amide, and polysaccharide moieties—that enable adsorption via multiple mechanisms: electrostatic interaction, hydrogen bonding, chelation, and redox reaction. These findings reinforce the multifunctional biosorptive potential of *S. brachiata* and support its applicability in the phytoremediation of nutrient and heavy metal contaminants in saline environments.

## 3. Materials and Methods

### 3.1. Collection of Plant Materials

Plants of *S. brachiata* at the late senescence stage were collected from Karaitivu, Puttalam, Northwestern Province, Sri Lanka (80°13′26.30″ N: 79°47′42.22″ E) in August 2024. Approval for sampling was obtained from the relevant authorities. The plant species was identified using standard taxonomic keys, and a specimen was submitted to the National Herbarium in Peradeniya, Sri Lanka, under the accession number R2/RJ1_A [71]. Seeds were harvested from the air-dried stems, germinated in coir pellets, and transferred to the pots with a coir:sand mixture (1:1) for experimental purposes. The plants were irrigated on alternate days by using reverse osmosis (RO) water.

### 3.2. Plant Acclimatization for the Phytoremediation Experiment

Pot trials were conducted in a greenhouse at Mihintale, Rajarata University of Sri Lanka (8°21′31″ N: 80°30′21″ E), from November to December 2024. Healthy, 2-month-old plants, with 7–8 cm in height and 4–5 primary branches, were grown in a hydroponic culture solution containing 100 mM NaCl and Albert nutrient solution for a 14-day acclimatization period under white light (350 µmol m^2^ s^−1^) with an 8/16 h day/night cycle at a temperature of 25 to 30 °C [72]. Nutrient supply to the plants was discontinued three days prior to the experiment, during which the plants were starved in a nutrient-deprived conditions [73]. The greenhouse experiment was a complete-randomized design with three different treatment concentrations and a control for each tested pollutant.

### 3.3. Determiantion of the Phytoremediation Efficiency of S. brachiata for Selected Nutrients and Metals

The phytoremediation efficiency of *S. brachiata* in removing selected nutrients (NO_3_^−^, PO_4_^3−^, and Ca^2+^) and heavy metals (Pb^2+^, Cr^6+^, and Cu^2+^) was evaluated against three different concentrations decided based on the tolerance limits for the discharge of wastewater effluents into coastal waters [74,75] (Table 7). In each concentration, 12 uniformly healthy plants of similar size (fresh weight of 0.9–1.2 g per plant) were individually exposed to the selected pollutants under saline conditions (100 mM NaCl) for a period of 14 days, following a completely randomized design (CRD) with 3 replicates, each containing 4 seedlings. The control plant group was maintained under the same conditions, without the addition of nutrients or metals [33]. The pH of all the solutions was maintained between 5.0 and 6.8.

A sample aliquot of 15 mL was collected at two-day intervals, centrifuged at 3000 rpm (Thermo Centra CL2, Thermo Fisher Scientific, Vantaa, Finland) for 10 min to remove larger particles, and analyzed for concentration using the standard analytical procedures listed in Table 7, over 14 days. Briefly, NO_3_^−^ and PO_4_^3−^ concentrations were determined using a UV–Vis spectrophotometer (Thermo Scientific Evolution 201, Thermo Fisher Scientific, Vantaa, Finland), following the sodium salicylate and molybdate ascorbic acid methods, respectively. The concentrations of Ca, Pb, Cr, and Cu were measured using inductively coupled plasma optical emission spectroscopy (ICP-OES, ICAP 7000 series, Thermo Fisher Scientific, Bremen, Germany) where the limits of detection were 0.01 mg/L for Ca and 0.001 mg/L for Pb, Cr, and Cu metals. The RO water acidified with ICP grade HNO_3_ was used for the blank samples. Individual single element standard solutions were used as the calibration standards. The removal efficiency (RE) of nutrients and metals was calculated as given in Equation (1) [33]:

RE = (Ci − Co)/Ci × 100(1)

where Ci is the initial concentration of the selected ion in the solution on day 1, and Co is its concentration on a given day

After 14 days of treatment, the plants were washed three times with deionized water, and the following growth parameters were measured: root length, shoot length, number of primary branches, total fresh weight, and dry weight. All the chemicals used in the study were of the highest purity available and of the analytical reagent grade (Supplementary Table S1).

### 3.4. Plant Sampling for the Biochemical Analysis

The plants were separated into belowground (roots) and aboveground (shoots, including stems and leaves) components. The shoots were then randomly divided into two subsets: one subset was used for biomass assessment by measuring fresh weight, followed by drying in a hot air oven at 70 °C until a constant weight was achieved [22]. The second subset was used for biochemical analysis, where fresh samples were rinsed with sterile distilled water and immediately stored at −20 °C until further processing.

### 3.5. Quantification of the Photosynthetic Pigment Content

A plant tissue sample of 0.1 g was washed three times with deionized water and blotted dry with tissue paper, and homogenized in 80% acetone. The homogenate was incubated in the dark for 6 h and then centrifuged at 10,000 rpm for 10 min. The absorbance of the resulting supernatant was measured at 663 nm and 645 nm, respectively, by using a UV–Vis spectrophotometer, and chlorophyll content was determined using Equations (2)–(4) [79].

Chlorophyll (a) = [(12.21 × A663) − (2.81 × A645)] × mL Acetone/mg(2)

Chlorophyll (b) = [(20.13 × A645) − (2.81× A663)] × mL Acetone/mg(3)

Chlorophyll (t) = [(20.2 × A645) + (8.02 × A663)] × mL Acetone/mg(4)

### 3.6. Determination of Proline Content

The free proline content was determined according to the method of Bates et al. [80], with minor modifications [22]. The absorbance of the liquid phase was measured using a UV–Vis spectrophotometer at 520 nm. L-proline was used as the standard, and the proline content in the fresh mass was calculated based on the standard calibration curve.

### 3.7. Enzyme Extraction

For enzyme extraction, 0.3 g of tissue was frozen in liquid nitrogen to prevent proteolytic activity and ground up with 3 mL of extraction buffer (0.1 M phosphate buffer, pH 7.5, and 0.5 mM EDTA). The homogenate was centrifuged at 15,000× *g* for 20 min, and the resulting supernatant was used for enzymatic assays. The total protein concentration was measured with the Bradford assay [81], and the extract was kept on ice for further use.

#### 3.7.1. Determination of Superoxide Dismutase Activity

The superoxide dismutase activity was estimated by the decrease in absorbance of a formazone complex, produced by the interaction of a superoxide radical and nitro-blue tetrazolium dye (NBT) [82]. The reaction mixture contained 13.33 mM methionine, 75 mM NBT, 0.1 mM EDTA, 50 mM sodium carbonate, and 5 mg of enzyme in a final volume of 3 mL reaction mixture. The reaction was initiated by adding 2 mM riboflavin, followed by incubation under a 15-W fluorescent lamp for 15 min. A complete reaction mixture without the enzyme, which produced the maximal color intensity, served as the control. The absorbance was recorded at 560 nm. One unit of SOD activity was defined as the amount of enzyme required to inhibit NBT reduction by 50%.

#### 3.7.2. Determination of Polyphenol Oxidase Activity 

Polyphenol oxidase (PPO) activity was determined by measuring the increase in absorbance at 420 nm using 4-methylcatechol as the substrate in a spectrophotometric assay. The reaction mixture, with a total volume of 3.0 mL, contained 100 mM of sodium phosphate buffer (pH 7.0), 5 mM of 4-methylcatechol, and 0.5 mL of crude enzyme extract, and the assay was conducted at room temperature. One unit of enzyme activity was defined as the amount of the enzyme that caused an absorbance change of 0.001 per minute [83].

#### 3.7.3. Determination of Catalase Activity

Catalase activity was measured according to Dhindsa et al. [82]. The assay mixture contained 50 mM of sodium phosphate buffer (pH 7.0), 12.5 mM of H_2_O_2_, and 5 mg of enzyme extract. Adding H_2_O_2_ began the reaction, and the decrease in absorbance was recorded for 1 min at 240 nm to obtain the difference in H_2_O_2_ decomposition. The enzyme activity was expressed in units of 1 mM of H_2_O_2_ decomposed per minute per milligram of protein.

#### 3.7.4. Determination of Peroxidase Activity

The peroxidase activity was determined spectrophotometrically by measuring the increase in absorbance at 420 nm caused by the oxidation of 4-methylcatechol (substrate) by H_2_O_2_. The reaction mixture, with a total volume of 3.0 mL, contained 100 mM of sodium phosphate buffer (pH 7.0), 5 mM of 4-methylcatechol, 5 mM of H_2_O_2,_ and 0.5 mL of crude enzyme extract, and the assay was conducted at room temperature. One unit of enzyme activity was defined as a 0.001 change in absorbance per minute under assay conditions [84].

### 3.8. Characterization of the Plant Samples by Using FTIR

After 14 days of exposure to the selected pollutants (NO_3_^−^, PO_4_^3−^, Ca^2+^, Pb^2+^, Cr^6+^, and Cu^2+^), whole plants of *S. brachiata* were collected, rinsed sequentially with RO water and deionized (DI) water, and oven-dried at 60 °C until a constant weight was achieved to minimize moisture interference and ensure spectral clarity. The dried plant samples, including both roots and shoots, were finely ground using a mortar and pestle. For each sample, 0.5 g of the dried plant powder was mixed with 100 mg of spectroscopic-grade potassium bromide (KBr), and the pellet was subjected to Fourier transform infrared (FTIR) spectroscopy analysis. The spectra were recorded in the mid-infrared range (4000–400 cm^−1^) using a PerkinElmer Spectrum TWO LITA spectrometer (Model L1600300, PerkinElmer, Waltham, MA, USA). This analysis was conducted to characterize the chemical constituents present in the plant material and to identify the functional groups potentially involved in the pollutant absorption mechanisms [85,86,87].

### 3.9. Statistical Analysis

Data from each treatment group (n = 3) were subjected to one-way analysis of variance (ANOVA) using SAS software version 9.4 (SAS Ondemand for Academics, SAS Institute Inc., Cary, NC, USA) to evaluate the effects of pollutant concentrations on plant growth and biochemical responses. When significant differences were detected (*p* < 0.05), Duncan’s Multiple Range Test (DMRT) was performed to determine pairwise differences among treatment means. The results are presented as means ± standard error (SE) in tables, while standard deviation (SD) values are shown as error bars in figures. FTIR spectral data were processed and visualized using OriginPro- version 9.9, 2022 (OriginLab Corporation, Northampton, MA, USA).

## 4. Conclusions

This study provides compelling evidence for the multifaceted phytoremediation potential of *S. brachiata*, a highly salt-tolerant halophyte, under controlled hydroponic conditions. The plant exhibited substantial removal efficiencies for both nutrients (NO_3_^−^, PO_4_^3−^, Ca^2+^) and metals of toxicological concern (Pb^2+^, Cr^6+^, Cu^2+^), with several pollutants, particularly Pb^2+^ and Cr^6+^, showing near-complete removal within just two days of exposure. Unlike the gradual uptake of nutrients, heavy metals were removed rapidly, suggesting distinct underlying mechanisms, such as biosorption and surface complexation, as opposed to physiological assimilation alone.

Biochemical analyses revealed robust stress adaptation responses, including significant increases in proline content and the upregulation of key antioxidant enzymes (CAT, SOD, POD, and PPO), particularly under heavy metal stress. The FTIR spectra corroborated these findings by identifying active binding sites, namely –OH, –NH, and –COO^−^ groups, that facilitated the adsorption of both anionic and cationic species. These biochemical and structural insights reinforce *S. brachiata*’s resilience and functional versatility in pollutant sequestration.

Importantly, the current findings fill a critical gap in the literature by quantifying heavy metal removal efficiencies by *Salicornia* species in hydroponic systems—a condition more representative of engineered wetlands and saline wastewater treatment units. The integration of physiological, biochemical, and spectroscopic analyses provides a holistic understanding of the plant’s pollutant response mechanisms.

In conclusion, *S. brachiata* emerges as a strong candidate for eco-sustainable remediation strategies in saline and polluted coastal environments. Its high tolerance, rapid uptake kinetics, and dual capacity for nutrient recovery and metal detoxification offer promise for its use in integrated aquaculture, wastewater management, and soil reclamation efforts. Future research should prioritize long-term field validation, explore pollutant interactions under mixed contamination scenarios, and consider growth stage and salinity level variations in pollutant uptake when evaluating the phytoremediation efficacy of *S. brachiata*.

## Figures and Tables

**Table 1:** Effect of NO_3_^−^ on different parameters on *S. brachiata* in hydroponic culture at 14 days.

Parameter	Control	50 mg/L	100 mg/L	200 mg/L
Fresh weight (g)	13.08 ± 1.00 ^d^	15.77 ± 1.33 ^c^	16.09 ± 1.93 ^b^	18.43 ± 1.23 ^a^
Shoot length (cm)	20.50 ± 0.59 ^c^	23.21 ± 0.49 ^b^	25.76 ± 0.69 ^a^	25.96 ± 0.48 ^a^
Number of lateral branches	31.41 ± 1.20 ^c^	35.08 ± 0.40 ^b^	37.25 ± 3.10 ^a^	38.33 ± 2.40 ^a^
Root length (cm)	2.59 ± 0.98 ^c^	6.25 ± 0.98 ^b^	7.25 ± 1.35 ^ab^	7.61 ± 1.19 ^ab^
Chlorophyll (mg/g)	2.94 ± 0.84 ^a^	1.62 ± 0.21 ^b^	0.96 ± 0.09 ^c^	0.77 ± 0.24 ^c^
Proline (µg/100 mg)	0.40 ± 0.10 ^d^	1.56 ± 0.23 ^c^	2.88 ± 0.22 ^b^	7.07 ± 0.31 ^a^
Catalase (U/mg/min)	1.42 ± 0.09 ^d^	28.22 ± 0.47 ^c^	55.27 ± 0.28 ^a^	55.61 ± 0.05 ^a^
SOD (U/mg/min)	0.04 ± 0.01 ^d^	20.57 ± 0.14 ^c^	24.78 ± 0.19 ^b^	36.60 ± 3.40 ^a^
POD (U/mg/min)	10.06 ± 0.32 ^c^	11.40 ± 1.20 ^c^	13.24 ± 2.30 ^b^	16.96 ± 1.51 ^a^
PPO (U/mg/min)	0.03 ± 0.01 ^d^	21.59 ± 0.21 ^c^	23.62 ± 1.12 ^b^	38.55 ± 1.43 ^a^

Means with similar letters within a row are not significantly different at *p* = 0.05.

**Table 2:** Effect of PO_4_^3−^ on different parameters on *S. brachiata* in hydroponic culture at 14 days.

Parameter	Control	5 mg/L	10 mg/L	15 mg/L
Fresh weight (g)	13.04 ± 2.52 ^c^	18.58 ± 1.74 ^b^	24.27 ± 0.53 ^a^	23.22 ± 2.61 ^a^
Shoot length (cm)	24.87 ± 1.37 ^b^	28.35 ± 0.44 ^a^	29.75 ± 1.34 ^a^	29.15 ± 0.79 ^a^
Number of lateral branches	22.21 ± 1.20 ^c^	33.08 ± 0.41 ^a^	33.25 ± 3.10 ^a^	31.13 ± 2.20 ^a^
Root length (cm)	2.59 ± 0.15 ^a^	3.18 ± 0.20 ^a^	3.80 ± 0.51 ^a^	3.93 ± 0.38 ^a^
Chlorophyll (mg/g)	7.40 ± 0.64 ^ab^	4.52 ± 0.32 ^b^	6.87 ± 1.12 ^ab^	8.9 ± 1.30 ^a^
Proline (µg/100 mg)	0.18 ± 0.03 ^b^	0.99 ± 0.01 ^a^	0.20 ± 0.10 ^b^	0.83 ± 0.20 ^a^
Catalase (U/mg/min)	2.21±1.20 ^a^	2.16 ± 0.40 ^a^	2.09 ± 0.01 ^a^	2.20 ± 1.42 ^a^
SOD (U/mg/min)	0.05 ± 0.01 ^a^	0.02 ± 0.01 ^a^	0.018 ± 0.01 ^a^	0.02 ± 0.01 ^a^
POD (U/mg/min)	2.21 ± 0.01 ^a^	2.11 ± 0.11 ^a^	2.34 ± 0.21 ^a^	2.45 ± 0.23 ^a^
PPO (U/mg/min)	0.052 ± 0.001 ^a^	0.024 ± 0.001 ^a^	0.018 ± 0.002 ^a^	0.026 ± 0.003 ^a^

Means with similar letters within a row are not significantly different at *p* = 0.05.

**Table 3:** Effect of Ca^2+^ on different parameters on *S. brachiata* in hydroponic culture at 14 days.

Parameter	Control	10 mg/L	50 mg/L	100 mg/L
Fresh weight (g)	16.04 ± 2.52 ^a^	16.28 ± 1.74 ^a^	14.27 ± 1.54 ^ab^	11.22 ± 1.63 ^b^
Shoot length (cm)	28.22 ± 1.37 ^a^	26.35 ± 0.41 ^ab^	24.15 ± 1.34 ^b^	22.11 ± 0.11 ^b^
Number of lateral branches	30.21 ± 1.2 ^ab^	28.08 ± 0.42 ^ab^	23.25 ± 3.13 ^b^	20.33 ± 2.42 ^c^
Root length (cm)	3.29 ± 0.52 ^a^	2.80 ± 0.20 ^a^	2.80 ± 0.51 ^a^	1.23 ± 0.12 ^b^
Chlorophyll (mg/g)	6.06 ± 0.44 ^a^	5.11 ± 0.65 ^a^	7.10 ± 1.93 ^a^	3.31 ± 0.29 ^b^
Proline (µg/100 mg)	2.93 ± 0.03 ^c^	5.45 ± 0.01 ^b^	7.26 ± 0.05 ^ab^	8.33 ± 0.02 ^a^
Catalase (U/mg/min)	4.13 ± 0.11 ^d^	19.02 ± 1.14 ^c^	55.59 ± 2.45 ^b^	82.56 ± 2.37 ^a^
SOD (U/mg/min)	3.56 ± 1.31 ^c^	12.87 ± 2.52 ^b^	17.53 ± 1.25 ^b^	20.64 ± 1.46 ^a^
POD (U/mg/min)	1.23 ± 0.01 ^d^	6.11 ± 1.56 ^c^	15.8 ± 2.17 ^b^	21.91 ± 0.42 ^a^
PPO (U/mg/min)	4.32 ± 1.12 ^c^	12.87 ± 2.46 ^b^	13.93 ± 1.51 ^b^	20.45 ± 3.27 ^a^

Means with similar letters within a row are not significantly different at *p* = 0.05.

**Table 4:** Effect of Pb^2+^ on different parameters on *S. brachiata* in hydroponic culture at 14 days.

Parameter	Control	0.5 mg/L	1 mg/L	5 mg/L
Fresh weight (g)	11.14 ± 1.50 ^a^	9.58 ± 1.74 ^b^	9.27 ± 0.5 ^b^	8.22 ± 2.6 ^b^
Shoot length (cm)	22.17 ± 1.21 ^a^	19.15 ± 1.41 ^a^	18.75 ± 2.32 ^ab^	11.15 ± 2.79 ^b^
Number of lateral branches	26.41 ± 1.20 ^a^	25.08 ± 0.4 ^ab^	23.25 ± 3.1 ^ab^	21.33 ± 2.4 ^b^
Root length (cm)	2.59 ± 0.15 ^a^	2.08 ± 0.20 ^a^	2.10 ± 0.51 ^a^	2.11 ± 0.38 ^a^
Chlorophyll (mg/g)	6.18 ± 0.86 ^c^	5.87 ± 0.23 ^bc^	5.45 ± 0.31 ^b^	3.97 ± 0.98 ^a^
Proline (µg/100 mg)	1.18 ± 0.03 ^c^	3.92 ± 0.20 ^b^	4.33 ± 0.18 ^ab^	5.18 ± 0.19 ^a^
Catalase (U/mg/min)	2.23 ± 1.03 ^c^	55.61 ± 2.14 ^b^	82.19 ± 3.45 ^a^	82.56 ± 2.11 ^a^
SOD (U/mg/min)	0.48 ± 0.12 ^b^	0.884 ± 0.113 ^a^	-	-
POD (U/mg/min)	2.23 ± 0.11 ^d^	55.61 ± 0.47 ^c^	73.31 ± 2.21 ^b^	95.05 ± 1.41 ^a^
PPO (U/mg/min)	0.044 ± 0.001 ^c^	0.128 ± 0.021 ^b^	1.323 ± 0.031 ^b^	2.411 ± 0.023 ^a^

Means with similar letters within a row are not significantly different at *p* = 0.05; ‘-’ Not Detected.

**Table 5:** Effect of Cr^6+^ on different parameters on *S. brachiata* in hydroponic culture at 14 days.

Parameter	Control	0.5 mg/L	1 mg/L	5 mg/L
Fresh weight (g)	09.02 ± 2.51 ^a^	8.58 ± 1.74 ^ab^	7.21 ± 0.51 ^ab^	5.11 ± 2.64 ^b^
Shoot length (cm)	22.82 ± 1.71 ^a^	21.35 ± 1.21 ^a^	19.75 ± 1.14 ^ab^	17.15 ± 0.99 ^b^
Number of lateral branches	36.41 ± 1.23 ^a^	35.01 ± 0.42 ^a^	33.15 ± 3.13 ^ab^	30.33 ± 2.42 ^b^
Root length (cm)	3.19 ± 0.15 ^a^	3.11 ± 0.20 ^ab^	2.80 ± 0.21 ^b^	2.13 ± 0.38 ^b^
Chlorophyll (mg/g)	8.34 ± 1.07 ^a^	6.31 ± 0.26 ^ab^	5.41 ± 0.57 ^b^	5.58 ± 0.21 ^b^
Proline (µg/100 mg)	0.11 ± 0.01 ^c^	0.42 ± 0.21 ^b^	0.54 ± 0.02 ^b^	0.71 ± 0.03 ^a^
Catalase (U/mg/min)	18.39 ± 0.1 ^d^	28.73 ± 0.23 ^c^	54.58 ± 2.1 ^b^	81.54 ± 2.1 ^a^
SOD (U/mg/min)	1.96 ± 1.21 ^d^	45.26 ± 2.37 ^c^	73.47 ± 1.77 ^b^	107.75 ± 1.68 ^a^
POD (U/mg/min)	7.79 ± 2.12 ^d^	29.66 ± 1.31 ^c^	55.74 ± 1.12 ^b^	81.28 ± 3.1 ^a^
PPO (U/mg/min)	3.34 ± 1.11 ^d^	30.10 ± 2.23 ^c^	32.92 ± 2.16 ^b^	31.51 ± 4.17 ^a^

Means with similar letters within a row are not significantly different at *p* = 0.05.

**Table 6:** Effect of Cu^2+^ on different parameters on *S. brachiata* in hydroponic culture at 14 days.

Parameter	Control	0.25 mg/L	0.5 mg/L	1 mg/L
Fresh weight (g)	10.04 ± 1.51 ^b^	11.18 ± 1.74 ^a^	11.17 ± 0.21 ^a^	11.12 ± 2.12 ^a^
Shoot length (cm)	27.87 ± 1.17 ^a^	25.35 ± 0.44 ^b^	24.75 ± 1.34 ^c^	23.15 ± 0.79 ^d^
Number of lateral branches	30.36 ± 1.23 ^a^	27.98 ± 0.42 ^b^	23.1 ± 3.11 ^d^	25.33 ± 2.44 ^c^
Root length (cm)	2.79 ± 0.15 ^a^	2.18 ± 0.20 ^c^	2.80 ± 0.51 ^a^	2.63 ± 0.38 ^b^
Chlorophyll (mg/g)	6.03 ± 1.53 ^a^	5.04 ± 0.03 ^b^	4.54 ± 0.43 ^c^	4.05 ± 0.31 ^d^
Proline (µg/100 mg)	1.17 ± 0.03 ^d^	2.79 ± 0.05 ^c^	3.12 ± 0.20 ^b^	7.41 ± 0.12 ^a^
Catalase (U/mg/min)	2.23 ± 0.21 ^d^	29.02 ± 4.12 ^c^	55.59 ± 1.32 ^b^	82.56 ± 1.43 ^a^
SOD (U/mg/min)	7.56 ± 2.45 ^c^	12.87 ± 2.14 ^b^	17.53 ± 1.96 ^a^	20.64 ± 2.12 ^a^
POD (U/mg/min)	2.26 ± 1.41 ^d^	44.78 ± 1.38 ^c^	82.19 ± 2.22 ^b^	87.91 ± 0.90 ^a^
PPO (U/mg/min)	1.19 ± 0.33 ^c^	12.96 ± 2.16 ^b^	14.06 ± 2.42 ^b^	20.6 ± 1.71 ^a^

Means with similar letters within a row are not significantly different at *p* = 0.05.

**Table 7:** Tested treatment conditions, chemical sources, concentrations tested (mg/L), and the standard analytical methods used for the analysis.

Treatment	Chemical Source	Concentrations Tested (mg/L)	Method	References
NO_3_^−^	KNO_3_	0, 50, 100, and 200	Sodium salicylate method	[76]
PO_4_^3−^	KH_2_PO_4_	0, 5, 10, and 15	Molybdate ascorbic acid method	[76]
Ca^2+^	Ca(NO_3_)_2_.4H_2_O	0, 10, 50, and 100	ICP-OES	[77,78]
Pb^2+^	Pb(NO_3_)_2_	0.0, 0.5, 1.0, and 5.0	ICP-OES	[77,78]
Cr^6+^	K_2_Cr_2_O_7_	0.0, 0.5, 1.0, and 5.0	ICP-OES	[77,78]
Cu^2+^	CuSO_4_.5H_2_O	0.0, 0.25, 0.5, and 1.0	ICP-OES	[77,78]

## Data Availability

Data are available within the article.

## Supplementary Materials

The following supporting information can be downloaded at https://www.mdpi.com/article/10.3390/plants14131963/s1: Table S1: Details of chemicals utilized, including source information. Table S2: Details of apparatus employed, including model and source information.

## Abbreviations

The following abbreviations are used in this manuscript:

ROS	Reverse Oxygen Species-scavenging
CAT	Catalase activity
POD	Peroxidase activity
PPO	Polyphenol Oxidase activity
EDTA	Ethylene Diammine Tetra Acetic Acid
NBT	Nitro-Blue Tetrazolium Dye
ICP-OES	Inductively Coupled Plasma Optical Emission Spectroscopy
FTIR	Fourier Transform Infrared Spectroscopy
