# Supplementary material for: Multifaceted Pollutant Removal by Salicornia brachiata: A Phytoremediation Approach
Source: Plants (Basel). 2025 Jun 26;14(13):1963. doi: 10.3390/plants14131963 (PMC12252013; doi:10.3390/plants14131963)
Supplement: Supplementary file 1 [file plants-14-01963-s001.zip › plants-3695843-supplementary.pdf]

Supplementary Materials

Manuscript Title: Multifaceted Pollutant Removal by *Salicornia brachiata*: A Phytoremediation Approach

Table S1: Details of chemicals utilized, including source information

**Chemical name**	**Manufacturer**	**City and country of origin**
Sodium Chloride	Sisco Research Laboratories Pvt. Ltd.	Maharashtra, India
KNO_3	Research-Lab Fine chem Industries	Mumbai, India
KH_2PO_4	HiMedia Laboratories Pvt. Ltd.	Maharashtra, India
$Ca(NO_3)_2.4H_2O$	HiMedia Laboratories Pvt. Ltd.	Maharashtra, India
$Pb(NO_3)_2$	HiMedia Laboratories Pvt. Ltd.	Maharashtra, India
$K_2Cr_2O_7$	HiMedia Laboratories Pvt. Ltd.	Maharashtra, India
$CuSO_4.5H_2O$	HiMedia Laboratories Pvt. Ltd.	Maharashtra, India
Acetone	Chem-Lab NV	Belgium
Sulfosalicylic acid	Central Drug House Pvt. Ltd	Delhi, India
Toluene	Research-Lab Fine chem Industries	Mumbai, India
Glacial Acetic acid	Research-Lab Fine chem Industries	Mumbai, India
L-Proline	Central Drug House Pvt. Ltd	Delhi, India
Sodium phosphate monobasic	Research-Lab Fine chem Industries	Mumbai, India
Sodium phosphate dibasic	Research-Lab Fine chem Industries	Mumbai, India
EDTA	Central Drug House Pvt. Ltd	Delhi, India
Catalase	Sigma-aldrich	St. Louis, USA
Peroxidase	Sigma-aldrich	St. Louis, USA
Superoxidase dismutase	Sigma-aldrich	St. Louis, USA
Polyphenol oxidase	Sigma-aldrich	St. Louis, USA
L- methonine	Sigma-aldrich	St. Louis, USA
Nitroblue tetrazolium Dye	Sigma-aldrich	St. Louis, USA
Sodium carbonate	Sisco Research Laboratories Pvt. Ltd.	Maharashtra, India
Riboflavin	Sigma-aldrich	St. Louis, USA
4-methylcatechol	Sigma-aldrich	St. Louis, USA
Hidrogen peroxide	Chem-Lab NV	Belgium
Selenium	Central Drug House Pvt. Ltd	Delhi, India
Lithium sulfate	Central Drug House Pvt. Ltd	Delhi, India
Nitric acid	Research-Lab Fine chem Industries	Mumbai, India
Sulfuric acid	Research-Lab Fine chem Industries	Mumbai, India
Hydrochloric acid	Research-Lab Fine chem Industries	Mumbai, India
Sodium potassium tartrate	Central Drug House Pvt. Ltd	Delhi, India
Ammonium molybdate Tetrahydrate	Central Drug House Pvt. Ltd	Delhi, India
Sodium Hydroxide	Central Drug House Pvt. Ltd	Delhi, India
Trichloroacetic acid	Central Drug House Pvt. Ltd	Delhi, India
Bovine serum albumin	HiMedia Laboratories Pvt. Ltd.	Maharashtra, India

Table S2: Details of apparatus employed, including model and source information

Apparatus	Model	Manufacturer	City and country of origin
UV-Vis spectrophotometer	Thermo scientific Multiskan GO	Thermo Fisher Scientific	Vantaa, Finland
High speed centrifuge	Thermo Centra CL2	Thermo Fisher Scientific	Vantaa, Finland
Oven	OF3-45HP	Jeio Tech	Korea
Incubator	Stuart SI600	Stuart Equipment	United Kingdom
ICP-OES (Inductively coupled plasma emission spectrophotometer	ICAP 7000 series	Thermo Fisher Scientific	Bremen, Germany
Fourier Transform Infrared (FTIR) spectroscopy	Spectrum TWO LITA	PerkinElmer	Waltham, Massachusetts, United States
